# Supplementary material for: A UMLS-based spell checker for natural language processing in vaccine safety
Source: BMC Med Inform Decis Mak. 2007 Feb 12;7:3. doi: 10.1186/1472-6947-7-3 (PMC1805499; doi:10.1186/1472-6947-7-3)
Supplement: Additional file 13 — RAP application directory. Sets up directory for RAP (RDF application for PHP) source code [file 1472-6947-7-3-S13.gz › rap/doc/phpdoc/utility/StatementIterator.html]

xml version="1.0" encoding="iso-8859-1"?


Docs For Class StatementIterator


## Class StatementIterator

Description

Description |
Methods (details)

Iterator for traversing models.

This class can be used for iterating forward and backward trough MemModels. It should be instanced using the getIterator() method of a MemModel.

History:

- 08-10-2004 : Class completely rewritten
- 03-29-2004 : Problems with adding and removing statements fixed.
- 02-21-2003 : First version of this class.

- access: public
- author: Daniel Westphal <at d-westphal dot de mail at d-westphal dot de>
- author: Chris Bizer <chris@bizer.de>
- version: V0.9.1

Located in /util/StatementIterator.php (line 26)

```
Object
   |
   --StatementIterator
```

Method Summary

Description |
Methods (details)

StatementIterator
StatementIterator
(object MemModel &$model)

statement
current
()

integer
getCurrentPosition
()

boolean
hasNext
()

boolean
hasPrevious
()

void
moveFirst
()

void
moveLast
()

void
moveTo
(integer $position)

statement
next
()

statement
previous
()

Methods

Description |
Methods (details)

Constructor StatementIterator (line 51)

Constructor

- access: public

StatementIterator

StatementIterator
(object MemModel &$model)

- object MemModel
  &$model

current (line 150)

Returns the current statement.

- return: or NULL if there is no current statement.
- access: public

statement

current
()

getCurrentPosition (line 208)

Returns the current position of the iterator.

- access: public

integer

getCurrentPosition
()

hasNext (line 62)

Returns TRUE if there are more statements.

- access: public

boolean

hasNext
()

hasPrevious (line 90)

Returns TRUE if the first statement has not been reached.

- access: public

boolean

hasPrevious
()

moveFirst (line 165)

Moves the pointer to the first statement.

- access: public

void

moveFirst
()

moveLast (line 175)

Moves the pointer to the last statement.

- access: public

void

moveLast
()

moveTo (line 187)

Moves the pointer to a specific statement.

If you set an off-bounds value, the position will be set to the last element

- access: public

void

moveTo
(integer $position)

- integer
  $position

next (line 106)

Returns the next statement.

- return: or NULL if there is no next statement.
- access: public

statement

next
()

previous (line 135)

Returns the previous statement.

- return: or NULL if there is no previous statement.
- access: public

statement

previous
()

#### Inherited Methods


Inherited From Object

> Object::toString()

Documentation generated on Thu, 7 Jul 2005 13:43:58 +0200 by phpDocumentor 1.3.0RC3
